# A complex inflammatory mix: chorioamnionitis, antenatal steroids and early postnatal budesonide

**DOI:** 10.1038/s41390-024-03219-y

**Published:** 2024-05-09

**Authors:** Prue M. Pereira-Fantini, David Tingay, Satyan Lakshminrusimha

**Affiliations:** 1 Department of Paediatrics, University of Melbourne, Melbourne, VIC Australia; 2 Neonatal Research, Murdoch Children’s Research Institute, Parkville, VIC Australia; 3 Department of Pediatrics, UC Davis Children’s Hospital, Sacramento, CA USA

**Fig. 1:**
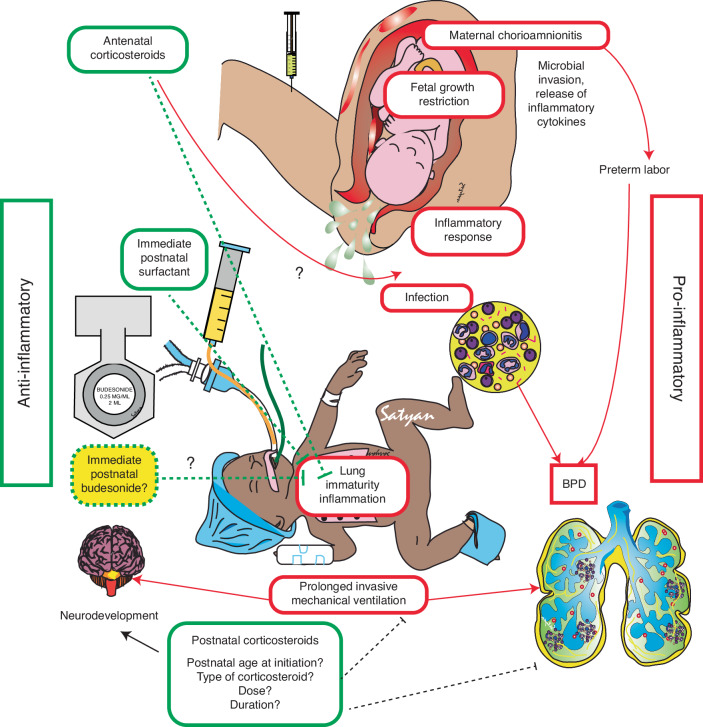
Inflammatory pathways associated with maternal chorioamnionitis and development of bronchopulmonary dysplasia (BPD). Fetal growth restriction and prenatal exposure to inflammatory mediators along with lung inflammation induced by prolonged invasive mechanical ventilation contribute to BPD in preterm infants. Anti-inflammatory agents such as antenatal corticosteroids and postnatal systemic corticosteroids induce lung maturity and reduce inflammation. Immediate intratracheal budesonide administered with surfactant has shown promising results in small trials and animal studies but results from multicenter trials are awaited. Green boxes – anti-inflammatory and red boxes are pro-inflammatory. Hyphenated lines suggest inhibition. Image courtesy Satyan Lakshminrusimha.

Bronchopulmonary dysplasia (BPD) remains the most common complication of premature birth, imposing a significant and potentially life-long burden on patients and their families.^1^ Chorioamnionitis and invasive mechanical ventilation are associated with lung inflammation and risk of BPD (Fig. 1).^2^ Reducing the risk of BPD begins well before birth. Optimising fetal growth and well-being generally, as well as the use of antenatal corticosteroids, in mothers expected to have a preterm delivery arguably has a bigger impact on lung protection than postnatal treatments provided in the neonatal intensive care units. Antenatal corticosteroids accelerate fetal lung maturation thereby decreasing the risk of perinatal death, neonatal death and respiratory distress syndrome.^3^ As the progression from early preterm lung disease to BPD is an inflammatory and immune mediated process, there has also been a longstanding interest in the potential for postnatal corticosteroids in preventing BPD. Although meta-analysis of 26 randomised controlled trials involving 4167 infants has shown early systemic corticosteroids reduce risk of BPD at 36 weeks postmenstrual age,^4^ the journey has not been a simple one with much uncertainty remaining. Specifically, the increased rate of abnormal neurological outcomes with early postnatal corticosteroids (<8 days of age) has appropriately limited translation.^5^ Balancing the risks and benefits of reducing BPD against those of corticosteroids in early preterm life remains challenging and clinicians are still seeking guidance on which preterm baby, and the optimal choice, timing and dose of corticosteroid to use.

The efficacy of postnatal corticosteroids in decreasing BPD in the presence of prenatal inflammation with chorioamnionitis is not known. Thus, the work of Hillman et al. in this issue of *Pediatric Research* is timely. In their study the authors assessed the respiratory benefits of combining antenatal corticosteroid administration with postnatal intratracheal corticosteroids and surfactant in the presence of antenatal inflammation. Pregnant sheep were given intra-amniotic lipopolysaccharide (LPS) to mimic chorioamnionitis and then randomised to IM betamethasone or saline and their offspring to surfactant with or without budesonide at 15 min after birth. For the first 15 min post-birth all lambs were mechanically ventilated using the same, and importantly, intentionally injurious ventilation strategy, followed by a gentler ventilation strategy for a further 3 h 45 min. Lambs were apnoeic throughout the study. They found that lambs receiving betamethasone with or without postnatal budesonide were more clinically stable than the saline control group with higher ventilation efficiency index, lower heart rate and pCO_2_, and improved lung compliance. The addition of budesonide to surfactant compared to surfactant alone in betamethasone exposed lambs resulted in further improvements in mean arterial pressure, pH, and pCO_2_. The finding that both approaches to corticosteroid exposure improved lung mechanics in this population of lambs is reassuring, expected and suggests that the described short-term benefit in humans and other preclinical populations was replicated.

Given the concerns with systemic corticosteroids in early postnatal life, budesonide is an attractive agent to modulate early neonatal lung disease. Unlike other potent corticosteroids it can be delivered directly to the lung and has an established role and safety profile in other paediatric populations such as asthma. Unfortunately clinical trials of budesonide alone for reducing BPD have not shown a benefit in the smallest preterm infants.^6^ The difficulty may partially relate to bioavailability and reliable dose delivery to the lungs of newly born premature infants. For this reason, delivering budesonide with a vector agent such as surfactant is attractive. Surfactant is a surface-active liquid complex of phospholipids and proteins that distributes to the alveoli, improves lung compliance and can carry other smaller agents well. As per their previous studies which examined budesonide pharmacokinetics in the setting of LPS-exposed ewes,^7^ LPS initially decreased plasma budesonide levels relative to maternal saline exposure, however the amount of budesonide remaining in the lung at 4 h was <3% of the initial dose in all groups, a finding also observed in previous studies by these investigators.^7,8^ The potential for systemic absorption of budesonide from the immature lung and distribution bypassing the liver (the primary site of first-pass metabolism of budesonide) warrants the need for further studies to assess the utility, safety and systemic toxicity of multiple-dose budesonide regimes.

Increase in the pulmonary gene expression of pro-inflammatory cytokines; IL1ϐ, IL6 and IL8 in response to ventilation has frequently been reported in preterm lamb models,^9,10^ however this study demonstrated a nuanced impact of betamethasone with or without budesonide treatment which was inflammatory pathway specific. Postnatal budesonide administration +/− antenatal betamethasone decreased gene expression of IL1β, IL8 and the chemokine MCP-1 gene expression suggesting some efficacy in dampening aspects of the inflammatory response. There was no impact on gene expression of the inflammatory acute phase mediators; IL6 and SAA3. TLR4, which has long been recognised as the sensing receptor for LPS,^11^ was increased in all lambs exposed to maternal LPS, confirming the utility of the maternal LPS-exposure model in fetal chorioamnionitis studies. The decrease in TLR4 with the combination of betamethasone and budesonide mirrors previous studies conducted with human airway epithelial cells.^12^ Of note, the study is limited to examining six a priori selected inflammatory genes and therefore may not reflect other inflammatory contributions such as complement activation and endothelial inflammatory mediation. Further studies using non-targeted approaches such as proteomics or largescale protein arrays, in which thousands of proteins can be assessed in a single sample, could further contextualise the findings of Hillman et al.

Chorioamnionitis increases the risk of preterm birth, and the complications arising from preterm birth. But concerns regarding the use of corticosteroids in infection states has led to decreased use in chorioamnionitis. In studies conducted in high-income countries, antenatal corticosteroids may be safe and reduce adverse neonatal outcomes.^13^ However, in low-and-middle-income countries (LMIC), antenatal corticosteroids may not reduce neonatal adverse outcomes in births <5^th^ percentile in weight (preterm or growth restricted neonates) and be associated with higher neonatal mortality in larger neonates.^14^ There is a potential for antenatal inflammation and infection to modify the impact of antenatal corticosteroids on neonatal outcomes.^15^ Hillman et al demonstrate that antenatal inflammation does not inhibit the functional efficacy of antenatal corticosteroids to enhance respiratory and haemodynamic stability. This does not mean that broader safety can be inferred. Intraamniotic injection of LPS to induce inflammation is a different model from active bacterial infection resulting in chorioamnionitis as might be common in LMICs. Similarly, it cannot be concluded that the combination of antenatal and early single dose budesonide will modulate longer-term lung health based on initial inflammatory changes alone. It is reassuring that there is a similar promotor effect from early corticosteroid exposure to the lungs after a pregnancy exposure to chorioamnionitis or not. Early budesonide with surfactant reduced lung inflammatory markers in the original clinical trial of Yeh and colleagues and was associated with a reduction in BPD in a population of very high-risk preterm infants.^16^ Larger, more pragmatic trials are ongoing or have recently been completed. Whilst the results of these trials are not yet known the preclinical work of Hillman and colleagues.^7–9^ indicates that understanding the outcomes in preterm infants exposed to chorioamnionitis maybe an important secondary outcome.

Preclinical studies of prematurity have well described limitations, including confounders in treatments, small sample sizes and inter-species differences, and have often not resulted in the expected translational benefits. Caution with regards to direct translation and generalisation equally apply to this study. Despite this caution it is worth reflecting on the role preclinical research has had in corticosteroids and prematurity. Antenatal corticosteroids are cheap, simple and effective, and standard of care. This has contributed to improvements in paediatric outcomes at a global scale for more than a generation. The initial proof of concept work was performed in sheep by Liggins and colleagues.^17^ This work and the role of postnatal corticosteroids was then advanced by Jobe and colleagues.^18–20^ The strength of the study of Hillman and colleagues is in considering how it adds another layer of knowledge to this large body of work. Without these programs the justification to clinical trials would not have been possible, and in turn preclinical science is useful in how we understand and interpret the biological plausibility of findings from clinical trials once completed. Preclinical research has a role in informing and guiding concepts to clinical evaluation but must be of high-quality, rigorous and, importantly, replicated.

Whilst we await the results of large clinical trials, preclinical studies that may assist in elucidating the multifactorial inflammatory events surrounding preterm birth will be essential for guiding our understanding of why, when and how of early budesonide using surfactant as a delivery vector in preterm lung disease. While we await the results of multicenter clinical trials of early intratracheal budesonide that include neurodevelopmental and respiratory follow-up, caution must be taken not to implement these therapies outside research protocols. Nonetheless in a preclinical research setting, the study suggests that antenatal betamethasone stabilises haemodynamics and compliance in the setting of chorioamnionitis, with postnatal budesonide/surfactant acting to decrease both LPS- and inflammatory responses.
